# The Effect of Stress Management Training on Hope in Hemodialysis Patients

**DOI:** 10.5539/gjhs.v8n7p165

**Published:** 2015-11-18

**Authors:** Farzad Poorgholami, Sareh Abdollahifard, Marzieh Zamani, Marzieh Kargar Jahromi, Zohreh Badiyepeymaie Jahromi

**Affiliations:** 1 Reaserch center for Non-Communicable Diseases, Jahrom University of Medical Sciences, Jahrom, Iran; 2 Departments of Nursing and Midwifery, Jahrom University of Medical Sciences, Jahrom, Iran; 3 Department of Nutrition, Jahrom University of Medical Sciences, Jahrom, Iran; 4 Departments of Nursing and Midwifery, Jahrom University of Medical Sciences, Jahrom, Iran

**Keywords:** hemodialysis patients, stress management training, hope

## Abstract

**Introduction:**

Chronic renal failure exposes patients to the risk of several complications, which will affect every aspect of patient’s life, and eventually his hope. This study aims to determine the effect of stress management group training on hope in hemodialysis patients.

**Method:**

In this quasi-experimental single-blind study, 50 patients with renal failure undergoing hemodialysis at Motahari Hospital in Jahrom were randomly divided into stress management training and control groups. Sampling was purposive, and patients in stress management training group received 60-minute in-person training by the researcher (in groups of 5 to 8 patients) before dialysis, over 5 sessions, lasting 8 weeks, and a researcher-made training booklet was made available to them in the first session. Patients in the control group received routine training given to all patients in hemodialysis department. Patients’ hope was recorded before and after intervention. Data collection tools included demographic details form, checklist of problems of hemodialysis patients and Miller hope scale (MHS). Data were analyzed in SPSS-18, using Chi-square, one-way analysis of variance, and paired t-test.

**Results:**

Fifty patients were studied in two groups of 25 each. No significant difference was observed between the two groups in terms of age, gender, or hope before intervention. After 8 weeks of training, hope reduced from 95.92±12.63 to 91.16±11.06 (P=0.404) in the control group, and increased from 97.24±11.16 to 170.96±7.99 (P=0.001) in the stress management training group. Significant differences were observed between the two groups in hope scores after the intervention.

**Conclusion:**

Stress management training by nurses significantly increased hope in hemodialysis patients. This low cost intervention can be used to improve hope in hemodialysis patients.

## 1. Introduction

As an irreversible and progressive loss of kidney function, chronic renal failure eventually causes the patient to need dialysis or transplantation (Longo, Fauci, & Kasper, 2011). The global growing trend of this disease is indicative of increasing number of patients undergoing alternative treatments such as kidney transplant, peritoneal dialysis, and hemodialysis (Glover, Banks, Carson, & Martin, 2011; Ferreira, & Silva Filho, 2011). According to statistics by Iran’s Center for Special Diseases and Kidney Foundation of Iran, the number of patients with chronic renal failure reached 24,000 by the end of 2008, of whom, 48.3% underwent hemodialysis, and this statistics increased by about 15% every year. Hemodialysis leads to increased life expectancy in patients, but has no effect on underlying renal disease (Kargar Jahromi, Javadpour, Taheri, & Poorgholami, 2016; Monfared, Safaei, Panahandeh, & Nemati, 2009).

End-stage renal disease is incurable and patients will require kidney transplantation for survival. Considering limitations of kidney transplant, many patients have to opt for dialysis. Although dialysis saves lives and increases longevity, it entails a wide range physical, mental, and socioeconomic problem for patients, which collectively affect their quality of life (Ebad, Sodani, Faghihi, & Hosseinpoor, 2009; Poorgholami Javadpour, Saadatmand, & Kargar Jahromi, 2016). Evidence suggests that hemodialysis is responsible for dramatic changes in quality of life of patients with chronic renal failure. In addition to several physiological changes, these patients are faced with many psychological pressures, and each in turn can impair their mentality and personality, such that the majority suffers behavioral problems and unadjusted stresses such as anxiety, depression, isolation, denial, and delusion. These problems affect their quality of life and hope, such that more problems mean lower quality of life and hope (Sabetghadam Poorgholami et al., 2016).

Hope is defined as process of thinking about personal goals, and motivation for achievement of goals (causal thinking), and methods of achieving goals (intrusive thinking) (Narimani, 2008). Hope is considered a cognitive goal-oriented process. Snider and Lopez (2005) believe highly hopeful people can create greater number of ways to achieve their goals, and are sufficiently motivated for reaching their desired outcome. On the contrary, people with little hope are neither likely to find possible solutions for their goals, nor are they highly motivated to achieve them (Folkman, 2013). There seems to be a mutual relationship between physical and psychological illness (Harrison, Hara, Pope, Young, & Rula, 2011). Despair in patients has been reported to cause loss of appetite, weight loss, and reduced positive coping behaviors including non-compliance with medication regimen, reduced learning, worrying and disability (Roberts, Johnson, & Keely, 2004). As an inner psychological source, hope is associated with future goals and expectations and can also affect individual’s behaviors. Hope is a psychological issue associated with self-care, adaptation to disease, and positive health behaviors (Folkman, 2013). In relation to adaptation to chronic diseases, Miller believes there is a relationship among hope, health and recovery. Miller also believes there is a direct relationship between sedentary life (which leads to inability to solve problems, organize goals or take the right action) and weakness, disability, loss of self-esteem, and despair, all of which ultimately lead to non-recovery, frustration and disappointment (Miller, 2007). Since renal failure is a chronic and incurable disease, these patients may feel disappointed due to reduced physical capabilities, and functional and social changes (Pasha & Amini, 2009).

Patient training is considered an important nursing role in all areas (including public awareness of hospital roles, cause and symptoms of diseases, treatment and self-care, etc.) (Noohi & Pouraboli, 2009). This role has been emphasized by the American Nursing Association (ANA) Standards of Clinical Nursing Practice (Safavi & Brazzy, 2006). Patient training has many benefits, including reduced healthcare costs, increased quality of care, and helping patients to achieve greater independence and self-sufficiency (Mobarak & Karami, 2006; Najafi Mehri, Vahedparast, Hafezi, Saghafi, Farsi, & Vahabi, 2008).

Since nurses spend longer time with hemodialysis patients compared to other medical team members (Pudner, 2005). They are the best to generate hope in patients, and can easily use non-medication therapy to enhance hope in patients (Forchuk & Reynolds, 2001). Providing non-medication measures by nurses (often with lower risk for patients) can increase hope, and can lead to cessation or reduction of drug use (Phipps, Monahan, Sands, Marek, & Neighbors, 2003). Using nursing interventions to increase hope is not in conflict with medical standards. One of such methods is teaching patients how to manage stress (Hossein Rezaei & Abbaszadeh, 2002). Stress management is referred to as a set of techniques used to reduce stress, or to increase the ability to cope with life stresses. This method is effective in psychosomatic illnesses, and even in diseases such as cancer and multiple sclerosis (MS) (Hasanvandi, Valizade, Mehrabizade Honarmand, & Mohammadesmaeel, 2013).

Despite all this and planning educational programs, in practice, patient training is less applied (Coates, 1999). In Iran, patient training program is also poor, and evidence suggests that patient training is either not implemented, or is implemented incompletely and irregularly (Mardanian Dehkordi, Salahshorian, Mohammad Alayha, & Hosseini, 2004).

Increased knowledge of stress management in hemodialysis patients can increase their performance, and increased stress management ability promises increased hope in these patients. Overcrowded hemodialysis department is highly stressful for patients, and inadequate nurse/patient ratio does not lend itself to receiving necessary training. Given all the above, and cultural, social, and economic differences in Iran (compared to other countries), this study aims to assess the effect of stress management training on hope in hemodialysis patients, so that a small step may be taken to abate patient suffering and reduce costs incurred to these patients.

## 2. Methods

### 2.1 Study Design

This case-control quasi-experimental study was approved by the Ethics Committee of Shiraz University of Medical Sciences, and registered at the Ministry of Health Center for Clinical Trials (code No IRCT2014042617440N1).

### 2.2 Study Setting and Sample

This study was conducted over 4 months from November 2014 to March 2015 in hemodialysis department of Motahari Hospital in Jahrom, with a sample size of 25 in each group (taking into account 10% withdrawal). Study population consisted of patients with advanced chronic renal failure attending hemodialysis department of Motahari Hospital in Jahrom in 2013-2014. Study participants were selected purposively and were then randomly divided into two groups using Random Allocation software. Sampling was conducted after obtaining necessary permissions and coordination with hemodialysis department authorities. Study inclusion criteria were the following: age between 18 and 65 years old, end stage renal disease, constantly receiving hemodialysis treatment for at least 6 months for once to three times per week lasting 3 to 4 hours each time, with no intention to leave Jahrom or to have kidney transplant in the course of intervention, receiving no formal hemodialysis training, no cognitive or psychological disorders, Farsi speaking with minimum literacy, and having a mobile phone. Study exclusion criteria included history of adverse events or experience in the recent 6 months, receiving antidepressants, admission for acute illness, and unwillingness to take part.

### 2.3 Data Collection

First, a questionnaire containing inclusion criteria were given to patients, and if they met the inclusion criteria, they were selected as participants, and written consent was obtained from them. Next, data were collected using a demographic questionnaire and Miller hope scale.

**The Questionnaire**

Miller hope scale was first used to assess hope in cardiac patients in America. Miller hope scale contains 48 items on hope and despair. Each item scores from 1 to 5 marks, adding up to a total score of 240, indicating maximum hope. Minimum hope is shown by minimum score of 48. Validity and reliability of this questionnaire and its suitability for assessment of hope in Iranian population were investigated in a study by Abdi and Asadi-Lari (2011) that found Cronbach’s alpha 80%, and confirmed it as the best tool for assessment of hope in Iranian patients (Abdi & Asadi Lari, 2011).

Content validity method was used to validate the questionnaires. After compilation based on literature, comments from nephrologists, urologists, psychiatrists, psychologists, nurses, and 5 members of study population were received and implemented. Reliability was assured with Cronbach’s alpha 80% found through completion of questionnaires by 10 patients in the study population. Prior to commencement, explanations were given to both groups about how to complete forms and questionnaires.

**Intervention**

Patient training group (stress management training) received 60-minute in-person training by the researcher (groups of 5 to 8 patients) before dialysis, over 5 sessions, lasting 8 weeks.

Patient training group also received a researcher-made booklet, in which explanations and advice were provided pertaining to the process of disease, the importance of hemodialysis, dietary regimen, limited liquid intake, daily weight control, physical activity, and control of vital signs, introduction to symptoms of underlying diseases, the importance of quitting smoking, stress management, and muscle relaxation. Patients in this group were also able to phone the researchers 24 hours a day in case of questions or problems. The control group received routine training provided in hemodialysis department, and did not participate in training sessions or receive educational booklet. Miller hope scale was again completed by the patients two months after completion of training. Study was performed in a single-blind format, so that participants had no knowledge of the training and control groups.

### 2.4 Data Analysis

Data were analyzed by SPSS-18 software using one-way analysis of variance and Chi-square tests at significance level of P<0.05.

## 3. Results

The majority of patients were about 50 years of age or younger. The mean age was 50.92±6.46 years in the stress management group, and 49.4±6.04 years in the control group. According to statistical tests, these showed no significant difference between the two groups. Chi-square test showed that both groups are similar in socio demographic characteristics. Table 1 shows some socio-demographic characteristics of the patients.

**Table 1:** Frequency distribution of the study units based on demographic variables in two groups

Group Features		Training	Control	P value

		Relative frequency	Relative frequency	
Sex	Female	57%	42%	0.40
	Male	43%	58%	
Marital status	Single	27%	16%	0.30
	Married	73%	84%	
Employment	Yes	41%	46%	0.43
	No	59%	54%	
Education level	Primary	3%	3%	0.62
	Junior high school	37%	39%	
	High school	56%	50%	
	Academic	4%	8%	
Hemodialysis frequency in a week	Twice	42%	38%	0.16
	Three times	58%	62%	

Before intervention, mean hope score was (97.24) in training group and (95.92) in control group and Chi-square test showed no significant difference between the two groups in this regard (P=0.404) (Table 2). After intervention, mean hope score was 170.96±7.99 in the stress management group, and 91.16±11.06 in the control group, with a significant difference between the two groups (P=0.001). Chi-square and ANOVA test showed a significant difference in mean hope score between the two groups (P=0.0001), and Chi-square test showed significant differences in a pair-wise comparison between training group and control group (P=0.0001).

**Table 2:** Mean hope score before and after intervention in hemodialysis patients

*Group* *Stages of treatment*	*Training group*	*Control group*	Paired t-test results

	Mean (SD)	Mean (SD)
Before intervention	97.24±11.6	95.92±12.63	NS
After intervention	170.96±7.99	91.16±11.06	P<0.001
Mean increase in hope score after intervention	73.72	4.76	P<0.001
Intra-group difference (independent t-test <0.001) NS			

After intervention, mean hope score reduced by 4.76. A significant difference was found in mean hope score before and after intervention in the intervention group (P<0.05), and after intervention, mean hope score increased by 73.72. Independent t-test showed no significant difference between the two groups in mean hope score before intervention (P=0.8). But, after intervention, the difference between the two groups was significant (P=0.001).

## 4. Discussion

The present study aimed to assess the effect of stress management training on the level of hope in hemodialysis patients. Descriptive data showed that trial and control groups matched in terms of age, gender, training, and duration of disease. According to the results, intervention was able to increase mean hope score in stress management training group compared to the control. Moreover, there was a significant difference between the two groups in terms of pretest and posttest mean scores (P<0.001). In other words, the difference between the two groups in hope scores was significant. Studies conducted on progressive renal failure in Iran so far have rather focused on patients’ quality of life, stress and coping mechanisms using quantitative or statistical methods, but stress management training and its effect on hope in hemodialysis patients in Iran had not been studied. Stress management training can reduce anxiety and depression, and increase the number of ways to cope with stress. Furthermore, it is a strategy for better control of the disease, reduction and prevention of physical and psychosocial complications. Stress management has been considered effective in many psychological disorders such as anxiety and depression, psychosomatic diseases, and even diseases such as cancer and multiple sclerosis. Studies by McGregor (2004) and Antoni (2006 and 2009) show that stress management training led to reduced anxiety, stress, and depression in women with breast cancer (McGregor et al., 2004; Antoni et al., 2006; Antoni, 2009). Some researchers consider stress management group training effective in reducing negative emotions and increasing a sense of self-efficacy and hope in chronic patients (Cobden, Niessen, Barr, Rutten, & Redekop, 2010). According to the results obtained, 5 sessions of stress management training led to improvement in mean hope score in the intervention group compared to the control. The present study results concur with those found by Hassanvandi et al. (Hasanvandi, Valizade, Mehrabizade Honarmand, & Mohammadesmaeel, 2013). And Hamid et al. regarding positive effect of stress management training program (Hamid, 2011). Results of a study by Shojaee et al. showed a significant improvement in mean hope score in the intervention group after intervention (Shojaee, Nehrir, Naderi, & Zareiyan, 2012), which agrees with the present study results. Researchers in nursing have long been investigating hope, and Fryback considers hope as one of the three main subjects in mental, emotional, and health of patients with end stage diseases. Some researchers in nursing even propose hope as a part of patient care process. Thus, nurses can inspire hope in patients by applying interventions with the above objectives. Quoting Lavaven, Tsay asserts that there is a direct relationship between taking responsibility for self-care and emotional-psychological states in patients undergoing maintenance hemodialysis (Tsay & Healstead, 2002). Eresk et al. reported the following factors as the most important nursing strategies for generating hope in patients: symptom management, making patients happy with occasional jesting, encouraging patients’ creative activities, minimizing social limitations, easy communication, reviewing past successes, organizing and correcting objectives, spending time with patient’s family, and emphasizing patient’s religious beliefs (Shojaee, Nehrir, Naderi, & Zareiyan, 2012). In the present study, patients that received stress management training had higher mean hope scores. Therefore, hope and stress can be considered as factors affecting outcomes of chronic diseases. All above studies concur with the present study regarding stress management training, and the results showed that stress management training led to better mean hope scores in the intervention group compared to the control (Narimani, 2008; Ebad Sodani, Faghihi, & Hosseinpoor, 2009). To improve hope in patients with chronic diseases, the majority of studies emphasize patient training and increased patient information, improvement in patient self-care performance, reinforcement of beliefs, and ultimately, all-out patient support. Other studies consider the following as interventions to increase hope in patients: patient information, improving self-care, social support programs, educational sessions, programs designed for patient discharge, programs that improve sense of being in control in patients, increased patient contact with medical team, and secure environment for expressing anxieties and problems (Barnason, Zimmerman, & Young, 2012; Moattari Ebrahimi, Sharifi, & Rouzbeh, 2012).

Due to the high prevalence of patients requiring hemodialysis, to prevent increased medical costs, and to enhance the process of recovery, it is necessary to use psychological therapies. Stress management and life skills training are considered effective in reducing stress, anxiety and depression in general population (Hasanvandi, Valizade, Mehrabizade Honarmand, & Mohammadesmaeel, 2013; Hamid, 2011). Therefore, providing such trainings can also be beneficial to hemodialysis patients. Also, to better provide hemodialysis patients with psychological health, stress management group training (or individual training if required) is beneficial, and will help reduce problems of these patients. Given adverse social, economic, and psychiatric effects of hemodialysis on family and community, it is recommended to use stress management strategies in hemodialysis patients.

Considering chronic nature of the disease in patients undergoing hemodialysis, despair is inevitable in these patients. On the other hand, the role of this internal factor cannot be overlooked in self-care in these patients.

## 5. Conclusion

The present study results showed that stress management training leads to increased hope in the case group. Finally, this strategy can also be recommended for inspiring hope in patients suffering other chronic diseases.
